# Assessment of Therapeutic Response in Pyogenic Vertebral Osteomyelitis Using ^18^F-FDG-PET/MRI

**DOI:** 10.3390/diagnostics10110916

**Published:** 2020-11-08

**Authors:** Ikchan Jeon, Eunjung Kong, Sang Woo Kim, Ihn Ho Cho, Cheol Pyo Hong

**Affiliations:** 1 Department of Neurosurgery, Yeungnam University College of Medicine, Daegu 42415, Korea; sw902@ynu.ac.kr; 2 Department of Nuclear Medicine, Yeungnam University College of Medicine, Daegu 42415, Korea; kongej@yu.ac.kr (E.K.); ihcho@med.yu.ac.kr (I.H.C.); 3 Department of Radiological Science, Catholic University of Daegu, Gyeongbuk 38430, Korea; chong@cu.ac.kr

**Keywords:** pyogenic, spine, therapeutic response, FDG-PET/MRI, SUV

## Abstract

Purpose: There is still no definite method to determine therapeutic response in pyogenic vertebral osteomyelitis (PVO). We analyzed the value of ^18^F-fluorodeoxyglucose positron emission tomography (FDG-PET) for assessing therapeutic response in PVO. Methods: This retrospective study included 53 patients (32 men and 21 women) with lumbar PVO. The results of clinical assessments for therapeutic response were divided into “Cured” (group C) and “Non-cured” (group NC). The differences in clinical and radiological features of PVO lesions between the two groups were analyzed using clinical data and simultaneous FDG-PET/magnetic resonance imaging (MRI) obtained at each clinical assessment. Results: Clinical assessments and FDG-PET/MRIs were performed at 41.89 ± 16.08 (21–91) days of parenteral antibiotic therapy. There were 39 patients in group C and 14 in group NC. Diagnostic accuracies (DAs) of FDG uptake intensity-based interpretation and C-reactive protein (CRP) for residual PVO were as follows (*p* < 0.01): 84.9% of the maximum standardized uptake value of PVO lesion (PvoSUV_max_), 86.8% of ΔPvoSUV_max_−NmlSUV_max_ (SUV_max_ of normal vertebra), 86.8% of ΔPvoSUV_max_−NmlSUV_mean_ (SUV_mean_ of normal vertebra), and 71.7% of CRP. DAs were better (92.5–94.3%) when applying FDG uptake intensity-based interpretation and CRP together. Under the FDG uptake distribution-based interpretation, FDG uptake was significantly limited to intervertebral structures in group C (*p* = 0.026). Conclusion: The interpretations of intensity and distribution of FDG uptake on FDG-PET are useful for detecting residual PVO in the assessment of therapeutic response of PVO. The combination of FDG-PET and CRP is expected to increase DA for detecting residual PVO.

## 1. Introduction

Unlike other infections, the symptoms of pyogenic vertebral osteomyelitis (PVO) are non-specific, and may not necessarily include fever [1,2]. Generally, before diagnosis, PVO would have already progressed, and vertebral and discal damages easily become severe with abscesses in the epidural space or paravertebral soft tissues [3]. Approximately 50% of PVO are treated with empirical antibiotics due to the culture being negative for causative bacteria [4]. PVO is usually treated conservatively with long-term antibiotics; a treatment duration of six to twelve weeks is recommended for patients without other complications [1,5,6,7]. A study that conducted under the same medical environment this study reported that an average duration of 8.0 ± 4.1 weeks of intravenous antibiotics administration was required to treat PVO [8]. A recent guideline recommended six weeks of parenteral or highly bioavailable oral antibiotics [1]. However, guidelines for treating PVO remain still ambiguous due to variability in causative bacteria and regional antibiotic resistance [5].

The assessment of therapeutic response has mainly been based on clinical symptoms and hematological inflammatory indices such as C-reactive protein (CRP) and erythrocyte sedimentation rate (ESR). It is still difficult to identify a therapeutic response and decide on when to discontinue antibiotics due to inconsistencies in clinical symptoms and hematological inflammatory indices. Generally, CRP decreases more quickly in patients presenting with clinical improvement and is more strongly associated with clinical symptoms than ESR and white blood cell (WBC) count [9]. Even with magnetic resonance imaging (MRI), it is difficult to distinguish between residual PVO lesions and granulation tissues resulting from tissue damages. Moreover, in cases demonstrating clinical recovery, MRI can show a worsened condition compared to previous images because tissue damage can take several months to resolve [10,11].

Studies related to the use of ^18^F-fluorodeoxyglucose positron emission tomography (FDG-PET) to assess therapeutic response in spinal infection have recently been introduced, and which is less affected by other conditions than hematological inflammatory indices. Although FDG-PET shows metabolic features of an infectious condition, its use is limited to visualizing anatomical changes. The application of simultaneous FDG-PET/MRI allows for greater anatomical resolution in addition to metabolic feature of FDG-PET in spine infection while minimizing temporal and spatial errors that occur during separate applications of FDG-PET and MRI [12,13,14]. However, most previous studies for assessing therapeutic response in spinal infection have been conducted with FDG-PET/computed tomography (CT) [15,16,17,18]. Some studies have been conducted on patients with mixed etiologies of spine infection. Moreover, there is a difference between the times of performing FDG-PET/CT and assessing therapeutic response, which is limited to determine the discontinuation of antibiotic therapy.

In this study, we analyzed the diagnostic value of FDG-PET for detecting residual lesion based on a simultaneous FDG-PET/MRI performed at the time of clinical assessment for therapeutic response after antibiotic therapy, and compared it with that of ESR, CRP, and MRI.

## 2. Materials and Methods

### 2.1. Patients and Data Collection

This retrospective study was conducted with clinical and radiological data of 77 patients (48 men and 29 women) from February 2017 to January 2020 in a single tertiary institution. They had clinical symptoms including fever, back pain, and/or neurological signs with specific MRI findings of lumbar PVO as a contiguous single lesion with/without positive cultures of PVO lesion and/or whole blood [19]. In the extent of PVO lesion, when a PVO lesion comprised an upper and a lower vertebrae centering infected disc with/without epidural, psoas, and paraspinal abscesses, it was defined as one level (e.g., if there were two infected discs, then three vertebrae were included centering on the two infected discs, this PVO lesion was considered two levels). We excluded patients with only paraspinal infection who did not have spondylodiscitis, tuberculous spondylodiscitis, tumors, bone infection at another site, trauma, concomitant severe medical problems, pregnancy, spinal instrument on PVO lesion, or age <20 years. The choice of parenteral antibiotics was decided according to the recommendation of infectious disease physicians. All patients underwent a follow-up period of at least six months after the discontinuation of parenteral antibiotic therapy.

All patients participated in this study with voluntary written informed consent to perform additional simultaneous FDG-PET/MRI in clinical assessment for therapeutic response. All the clinical and radiological data were obtained and reviewed under the approval of the institutional review board (Yeungnam University Hospital, 2016-12-019-013, and 22 December 2016).

### 2.2. Clinical Assessment for Therapeutic Response

All patients underwent clinical assessment for therapeutic response (twice a week) based on clinical symptoms and hematological inflammatory indices including CRP (normal range: <0.5 mg/dL) and ESR (normal range: <25 mm/h) after at least three weeks of parenteral antibiotic therapy. Visual analog scale (VAS) was used to measure back pain on a scale of 0–10 with 0 representing no pain and 10 representing maximum pain. When “Cured” was determined in clinical assessment for therapeutic response, parenteral antibiotic therapy discontinued. The condition of being “Cured” was defined as the absence of fever and improved clinical symptoms and CRP, which continues during a follow-up period without any other evidence of residual or recurrence. The condition of being “Non-cured” was defined as sustained or re-aggravation of clinical symptoms with presence of fever or identification of causative bacteria from blood/PVO lesion even after sufficient antibiotic therapy (treatment failure; antibiotic therapy was continued); or relapse of clinical symptoms with presence of fever, elevated hematological inflammatory indices, and/or identification of causative bacteria during the follow-up period of at least 6 months after discontinuation of parenteral antibiotic therapy under the determination of “Cured” (recurrence) [20]. Some of “Cured” in the initial clinical assessment for therapeutic response were finally changed to “Non-cured” with identification of residual or recurrence during the follow-up period. The patients were divided into “Cured” (group C) or “Non-cured” (group NC) based on the final results. Simultaneous FDG-PET/MRIs were performed once per patient when clinical assessment determined with “Cured” and “Non-cured (treatment failure)”, and classified into groups C and NC based on the final results. Simultaneous FDG-PET/MRI had no effect on the process of clinical assessment and was analyzed with clinical data under retrospective study design.

### 2.3. Intensity-Based Interpretation: FDG Uptake on FDG-PET

The differences in the intensity of FDG uptake between groups C and NC were analyzed. The measurement parameters for the intensity of FDG uptake included the maximum standardized uptake value of PVO lesion (PvoSUV_max_), difference between PvoSUV_max_ and SUV_max_ of normal vertebra (NmlSUV_max_) (ΔPvoSUV_max_−NmlSUV_max_), and difference between PvoSUV_max_ and mean standardized uptake value of normal vertebra (NmlSUV_mean_) (ΔPvoSUV_max_−NmlSUV_mean_). We analyzed the cut-off value, sensitivity, specificity, positive predictive value (PPV), negative predictive value (NPV), and diagnostic accuracy (DA) of PvoSUV_max_, ΔPvoSUV_max_−RefSUV_max_, and ΔPvoSUV_max_−NmlSUV_mean_ for detecting residual PVO.

In each case, a volumetric region of interest (ROI) was drawn over the entire area of abnormal uptake in the PVO lesion viewed on the axial and sagittal fused FDG-PET/MRI on the PET/MRI workstation (Syngo.via, Siemens Healthcare). PvoSUV_max_ was calculated for each ROI and recorded automatically on the workstation. FDG uptake of the normal vertebra located two segments away from the PVO lesion was measured as reference for non-infectious marrow activity. A volume ROI as large as possible inside the vertebra body without fracture and other deformity was established.

### 2.4. ESR and CRP

The differences in ESR and CRP between groups C and NC were analyzed. We analyzed cut-off value, sensitivity, specificity, PPV, NPV, and DA of ESR and CRP for detecting residual PVO, and compared with that of PvoSUV_max_, ΔPvoSUV_max_−NmlSUV_max_, and ΔPvoSUV_max_−NmlSUV_mean_. We also obtained sensitivity, specificity, PPV, NPV, and DA when applying CRP and FDG-PET together with using cut-off values of CRP and each parameters of the intensity-based FDG uptake including PvoSUV_max_, ΔPvoSUV_max_−NmlSUV_max_, and ΔPvoSUV_max_−NmlSUV_mean_ for detecting residual PVO.

### 2.5. Distribution-Based Interpretation: FDG Uptake on FDG-PET and Contrast Enhancement/High Signal Intensity on MRI

Imaging data from the simultaneous FDG-PET/MRIs were analyzed by two nuclear medicine physicians with over ten years of clinical experience in accordance with the following distribution-based interpretation criteria presented by Yu et al. [3] without prior knowledge of the patients’ clinical status. Distributions of FDG uptake on FDG-PET, contrast enhancement on T1-weighted contrast MRI (T1C), and high signal intensity on T2-weighted fat saturation MRI (T2FS) on PVO lesion were used for distribution-based interpretation.

1. Grade I: activity on bone, soft tissue, and epidural space with lower or similar intensity than the reference.
2. Grade II: limited activity on the margin or bulk of a destroyed disc and endplates rather than the bone, soft tissue, and epidural space with overall higher intensity than the reference.
3. Grade III: significantly increased activity on overall bone and soft tissue than the reference.

### 2.6. Simultaneous PET/MRI Data Acquisition

Patients fasted for at least 6-h beforehand and blood glucose levels were required to be <8.9 mmol/L before the injection of FDG (3.7 MBq/kg). Simultaneous spinal FDG-PET/MRI (Biograph mMR; Siemens Healthcare, Erlangen, Germany) acquisition was initiated 60 min after tracer injection and patients were scanned in one–two bed positions using an approved surface coil. FDG-PET data acquisition occurred over 20 min, and MRI data were simultaneously obtained using the predetermined sequence protocol in Table 1. A three-dimensional ordered subsets expectation maximization iterative reconstruction (OSEM-IR) algorithm was applied with three iterations and 21 subsets for FDG-PET data. A 172 × 172 matrix was used.

### 2.7. Statistical Analysis

Receiver operating characteristic (ROC) analysis was used to determine cut-off values of PvoSUV_max_, ΔPvoSUV_max_−RefSUV_max_, ΔPvoSUV_max_−RefSUV_mean_, ESR, and CRP for detecting residual PVO. Based on the confirmed cut-off values, sensitivity, specificity, PPV, NPV, and DA were analyzed. Student’s *t*-test for parametric continuous variables was used to compare two population means. Chi-squared test was used to assess the relationship between categorical variables. Statistical analyses were carried out using SPSS version 25.0 software (SPSS Inc., Chicago, IL, USA), and *p*-values < 0.05 were considered statistically significant.

## 3. Results

### 3.1. Patients and Clinical Assessment

Twenty-four among the 77 patients were excluded for the following reasons: follow-up loss or withdrawal of participation (*n* = 6), back muscle abscess without spondylodiscitis (*n* = 5), and misdiagnosis (*n* = 10, three of tuberculous spondylodiscitis, three of degenerative change, two of trauma, and two of ankylosing spondylitis), and spinal screw within PVO lesion (*n* = 3). The final analyses were performed on 53 patients (32 males and 21 females) with a mean age of 66.28 ± 11.48 (31–85) years. The patients were followed up for 12.32 ± 7.45 (6–35) months after discontinuation of parenteral antibiotic therapy. Fifty-three of simultaneous FDG-PET/MRIs were performed during the clinical assessments for therapeutic response at 41.89 ± 16.08 (21–91) days of parenteral antibiotic therapy. The final results of therapeutic response were consisted with 39 of group C and 14 of group NC (ten of treatment failure and four of relapse). There was no statistically significant difference in the timing of clinical assessment and simultaneous PET/MRI between the two groups (41.26 ± 16.29 (21–91) vs. 43.64 ± 15.95 (21–75) days of parenteral antibiotic therapy, *p* = 0.638). Detailed data including cause of PVO, comorbidity, initial clinical symptoms, and initial radiological features are presented in Table 2.

### 3.2. Microorganisms and Antibiotics

The rate of causative bacterial identification was 54.7% (29/53) under blood and/or PVO tissue culture, and there was no statistically significant difference between the two groups (20/39 vs. 9/14, *p* = 0.143). The main causative bacteria identified was methicillin-sensitive *Staphylococcus aureus* (MSSA). The mean duration of parenteral antibiotic therapy for treatment was 45.21 ± 18.45 (21–89) days. There was no statistically significant difference in the duration of parenteral antibiotic therapy between groups C and NC (42.33 ± 17.17 (21–89) vs. 53.21 ± 20.13 (23–88) days, *p* = 0.058). Vancomycin was required most commonly as the final effective parenteral antibiotics in 37.7% (20/53) of the patients. Detailed data are presented in Table 3.

### 3.3. ESR, CRP, and VAS

There were no statistically significant differences in the initial ESR, CRP, and VAS between groups C and NC. ESR when assessing therapeutic response showed no statistically significant difference between the two groups; however, ESR in group C improved with statistical significance after antibiotic therapy (*p* < 0.05). CRP and VAS showed statistically significant differences when assessing therapeutic response between the two groups, and significant improvements were noted in both groups after antibiotic therapy (*p* < 0.05). When 0.86 mg/dL was used as the cut-off value of CRP for residual PVO, the area under the curve (AUC) was 0.772 (*p* = 0.003). The sensitivity, specificity, PPV, NPV, and DA were 85.7%, 66.7%, 48.0%, 92.9%, and 71.7%, respectively. However, ESR showed no statistical significance in ROC analysis. Detailed data are presented in Table 4 and Table 5.

### 3.4. Intensity-Based Interpretation: FDG Uptake on FDG-PET

There were statistically significant differences in PvoSUV_max_ (*p* = 0.002), ΔPvoSUV_max_−NmlSUV_max_ (*p* = 0.013), and Δ PvoSUV_max_−NmlSUV_mean_ (*p* = 0.011) when assessing therapeutic response between groups C and NC. When 6.44 was used as the cut-off value of PvoSUV_max_ for residual PVO, the AUC was 0.833 (*p* < 0.001). The sensitivity, specificity, PPV, NPV, and DA were 71.4%, 89.7%, 71.4%, 89.7%, and 84.9%, respectively. When 4.50 used as the cut-off value of ΔPvoSUV_max_−NmlSUV_max_ for residual PVO, the AUC was 0.766 (*p* = 0.003). The sensitivity, specificity, PPV, NPV, and DA were 64.3%, 94.9%, 81.8%, 88.1%, and 86.8%, respectively. When 4.84 was used as the cut-off value of ΔPvoSUV_max_−NmlSUV_mean_ for residual PVO, the AUC was 0.773 (*p* = 0.003). The sensitivity, specificity, PPV, NPV, and DA were 64.3%, 94.9%, 81.8%, 88.1%, and 86.8%, respectively. Detailed data are presented in Table 4 and Table 5.

### 3.5. Combination of CRP and Intensity-Based Interpretation of FDG Uptake on FDG-PET

When the cut-off values of CRP and parameters of the intensity-based FDG uptake for detecting residual PVO are applied together, the sensitivity, specificity, PPV, NPV, and DA were 85.7%, 94.9%, 85.7%, 94.9%, and 92.5% in the combination of CRP and PvoSUV_max_; 85.7%, 97.4%, 92.3%, 95.0%, and 94.3% in the combination of CRP and ΔPvoSUV_max_−NmlSUV_max_; and 85.7%, 97.4%, 92.3%, 95.0%, and 94.3% in the combination of CRP and ΔPvoSUV_max_−NmlSUV_mean_, respectively. Detailed data are presented in Table 5.

### 3.6. Distribution-Based Interpretation: FDG Uptake on FDG-PET and Contrast Enhancement/high Signal Intensity on MRI

There were statistically significant differences in distributions of FDG uptake on FDG-PET (*p* = 0.026) and high signal intensity on T2FS (*p* = 0.022) between groups C and NC with a predominant frequency of Grade II in group C than group NC. However, TIC showed no statistically significant difference between the two groups. Detailed data are presented in Table 6.

## 4. Discussion

Although the guidelines for the use of antibiotics in PVO have not yet been clearly established, the minimum period of parenteral antibiotic therapy for PVO as proposed in the literature is 2–4 weeks [8,19,21]. In this study, the minimum duration of antibiotics was set to three weeks in accordance with previous studies that recommended short treatment duration [7,21]. Kim et al. [5] reported that the causative bacteria were identified only in half of all patients, with *S. aureus* as the most common PVO pathogen, showing methicillin resistance in 43.0% cases. We considered vancomycin as the first option of empirical antibiotics in patients with critical conditions or procedure-related PVO in our study. Vancomycin was recommended in culture-negative patients whose symptoms failed to alleviate or deteriorated further following the initial administration of antibiotics for MSSA. Consequently, vancomycin showed the highest efficacy as the final parenteral antibiotic at 37.7%, which was similar to the previously mentioned incidence of MRSA.

Because FDG-PET findings are less affected by other conditions, which can be a valuable independent method in the assessment of therapeutic response in PVO. To understand the differences in FDG uptake depending on the therapeutic response, it is important to identify the pathophysiological characteristics of each phases of osteomyelitis. In the early phase of osteomyelitis, activated neutrophil accumulation with increased vascular permeability, known as the respiratory burst, is observed; it uses greater amounts of glucose as the main energy source for chemotaxis and phagocytosis [22,23]. The transport of FDG across the cellular membrane is mediated by the glucose transporter, which is more abundantly found on the cell membrane of activated inflammatory cells and leads to increased FDG uptake in the acute phase [23]. In the chronic or recovery phase, however, lymphocytes are predominant, followed by plasma cells, histiocytes, and some polymorphonuclear leucocytes [24]. There is formation of fibroses and granulation tissues, new bone, and dilated blood vessels as well as fatty change around the foci of inflammation and bone marrow [24]. These findings show a decrease in FDG uptake and vascular permeability, it differs from the findings of early phase which shows a predominance of activated inflammatory cells.

**Figure 1:**
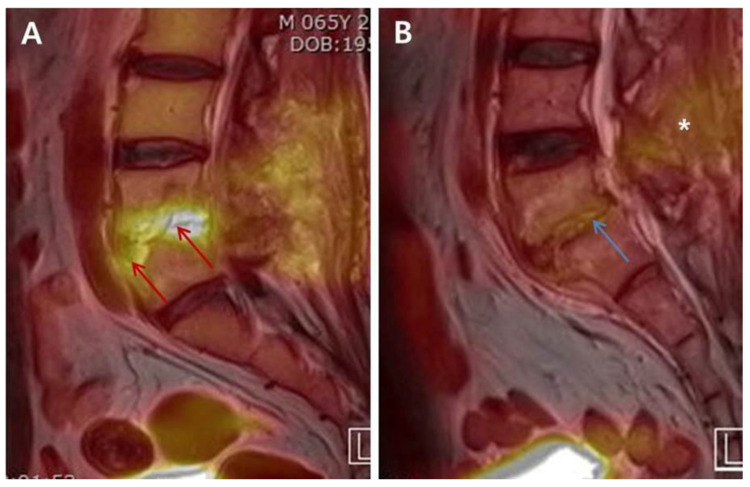
The differences in the intensity and distribution of FDG uptake between “Non-cured” and “Cured” simultaneous FDG-PET/MRIs. A 65-year-old male patient diagnosed with postoperative PVO was treated with 1st cephalosporin under the identification of MSSA. The clinical assessment on 21st days of parenteral antibiotic therapy showed sustained back pain, intermittent mild fever, and ESR/CRP 120/2.84. Simultaneous FDG-PET/MRI of “Non-cured” revealed an increased FDG uptake (red arrow; SUV_max_ 6.06; grade III of distribution-based interpretation criteria) on the anterior portion of L5 body and disc of L4–L5 (**A**). A 58-year-old male patient diagnosed with postoperative PVO presenting culture-negative was treated with vancomycin. The patient showed improved back pain, no fever, and ESR/CRP 61/0.343 in the clinical assessment on 45th days. FDG uptake decreased overall and limited on disc and endplates (blue arrow; SUV_max_ 3.12; Grade II) on simultaneous FDG-PET/MRI of “Cured”. There is also decrease of FDG uptake on posterior structures including back muscle layer (white asterisk) (**B**). Abbreviations: FDG = ^18^F-fluorodeoxyglucose; PVO = pyogenic vertebral osteomyelitis; MSSA = methicillin-sensitive *Staphylococcus aureus*; ESR = erythrocyte sedimentation rate (mm/h, normal range: <25 mm/h); CRP = C-reactive protein (mg/dL, normal range: <0.5 mg/dL); FDG-PET/MRI = ^18^F-fluorodeoxyglucose positron emission tomography/magnetic resonance imaging; SUV_max_ = maximum standardized uptake value of FDG.

In the distribution-based interpretation on FDG-PET, we identified a statistically significantly higher frequency of Grade II in group C compared with a predominant grade III in group NC (Figure 1). There may be mechanical stress caused by the patient’s activity in addition to the formation of granulation tissues on intervertebral structures during the recovery phase [16]; these can lead to the sustained increase of FDG uptake, thus indicating a Grade II pattern even after successful treatment and can lead to confusion in distinguishing the condition from residual PVO. However, we did not identify Grade I pattern in either group due to the timing of simultaneous FDG-PET/MRIs, which were performed at a relatively earlier time than previous studies did [3,17,18]. We expect Grade I pattern of FDG-PET to be obtained after a considerable amount of time has passed since the completion of antibiotic therapy. However, to minimize the duration of antibiotic therapy, earlier and accurate judgment of the therapeutic response is essential before the typical Grade I pattern appears. For experimental purposes, we adopted simultaneous FDG-PET/MRI to potentially identify a specific MRI sequence showing a similar pattern as FDG-PET based on the abovementioned pathophysiological characteristics associated with each phase of osteomyelitis. Unfortunately, unlike FDG-PET, MRI showed predominant incidence of Grade III in both groups even after successful treatment; however, T2FS showed Grade II pattern more frequently in group C than T1C did. Our results re-confirmed that extensive edema and contrast enhancement continued on MRI for a considerably long time even in cured PVO cases, which indicates the limitation of MRI in the early assessment of therapeutic response in PVO.

The stage of PVO at the time of diagnosis varies among the patients. If PVO is detected in the early stage, subsequent imaging studies can show a more deteriorated PVO lesion even under favorable progress of antibiotic therapy. Therefore, the assessment of therapeutic response by comparing with initial images as the reference can lead to inaccurate interpretation due to its wide variation among the patients. In addition, we sometimes cannot obtain initial images of PVO due to severe pain or unstable hemodynamic conditions. For these reasons, we adopted Δ PvoSUV_max_-NmlSUV_max_ and Δ PvoSUV_max_-NmlSUV_mean_ in the intensity-based interpretation, which use the value of FDG uptake of normal vertebrae as the reference instead of comparing with the value of pre-treatment PVO lesion on the initial image. These also enable a more accurate comparison between normal vertebrae and PVO lesion under the overall changed bone marrow condition after long-term antibiotic therapy. From these points of view, intensity-based interpretation may be more useful than distribution-based interpretation for assessing therapeutic response and determining discontinuation of antibiotic therapy. In particular, we observed overall advanced diagnostic values with >90% diagnostic accuracy when intensity-based interpretation was combined with CRP by addressing the limitations of the higher false-positive rate in CRP and false-negative rates in the parameters of intensity-based interpretation.

Despite the abovementioned advantages of FDG-PET, we need to consider that CRP is still widely used as an important method due to its cost-effectiveness and ease of use for examination when compared with the markedly higher costs associated with examination and the FDG-PET equipment. Considering the advantage that FDG-PET is less affected by other conditions than CRP, we recommend using FDG-PET when there are inconsistencies between clinical symptoms and CRP level which cause difficulties in determining the therapeutic response and discontinuation of antibiotic therapy. Additionally, we need to investigate the usefulness of MRI for assessing therapeutic response in PVO. In this study, the distribution pattern showed by T2FS was more similar to FDG-PET than to T1C. Given the aforementioned role of activated inflammatory cells in the FDG uptake, it is also known to produce exudate and increase vascular permeability during the respiratory burst activation in the acute phase of osteomyelitis [22,23]. Based on these features of activated inflammatory cells in the acute phase, we can consider the possibility of a relationship between high signal intensity, which indicates edematous change, on T2FS and FDG uptake on FDG-PET. Further prospective studies with many participants are required to identify the usefulness of T2FS as the MRI sequence presenting a similar pattern as FDG-PET.

## 5. Conclusions

The interpretations of intensity and distribution of FDG uptake on FDG-PET are useful for detecting residual PVO in the assessment of therapeutic response of PVO. In particular, the combination of FDG-PET and CRP is expected to increase DA for detecting residual PVO. However, we herein re-confirmed the limitation of MRI in the early assessment of therapeutic response of PVO, and further research based on the pathophysiological characteristics of PVO is required to advance the role of MRI.

## Figures and Tables

**Table 1:** Technical parameters for MRI sequences of integrated FDG-PET/MRI in this study.

Sequence	Image Plane	Contrast	TR (ms)	TE (ms)	Slice Thickness (mm)	Matrix Size (mm^2^)	GRAPA	FOV (mm)	Protocol Time (min)
3D VIBE ^a^	coronal	-	3.6	TE1 1.23 TE2 2.46	3.12	172 × 172	2	500	0:19
T2 TSE with fat saturation	sagittal	+ ^b^	4360	97	3.0	269 × 384	2	350	2:37
HASTE with fat saturation	axial	-	1000	69	3.0	180 × 320	2	400	1:05
T1 TSE	sagittal	-	634	9.1	3.0	314 × 448	2	350	2:34
T1 TSE with fat saturation	sagittal	-	668	8.8	3.0	260 × 384	2	350	2:04
T1 TSE with fat saturation	axial	-	683	10	2.6	218 × 256	2	200–250 ^c^	3:22

TE, echo time; TR, repetition time; GRAPA, generalized autocalibrating partially parallel acquisition; FOV, field of view; VIBE, volumetric interpolated breath-hold examination; TSE, turbo spin echo; HASTE, half-Fourier acquisition single shout turbo spine-echo; AC, attenuation correction; ^a^, Dixon-based attenuation correction; ^b^, Gadoterate meglumin (Dotarem, Guerbet, Aulnay-sous-Bois, France) 0.15 mL/kg with a rate of 1 mL/s; ^c^, according to lesion extent.

**Table 2:** Demographic and clinical data.

Factors	Group C (*n* = 39)	Group NC (*n* = 14)	Total
Age, years	65.97 ± 12.30 (31–83)	67.14 ± 9.16 (51–85)	66.28 ± 11.48 (31–85)
Sex (Male/Female)	26/13	6/8	32/21
Cause of PVO			
Spontaneous	18/39 (46.2%)	2/14 (14.3%)	20/53 (37.7%)
Procedure-related	21/39 (53.8%)	12/14 (85.7%)	33/53 (62.3%)
Injection or acupuncture	17/21 (80.9%)	7/12 (58.3%)	24/33 (72.7%)
Operation *	4/21 (19.0%)	5/12 (41.7%)	9/33 (27.3%)
Comorbidity			
Diabetes mellitus	14/39 (35.9%)	4/14 (28.6%)	18/53 (34.0%)
Hypertension	21/39 (53.8%)	6/14 (42.9%)	27/53 (50.9%)
Hemodialysis	2/39 (5.2%)	0/14 (0.0%)	2/53 (3.8%)
Cerebrovascular disease	3/39 (7.7%)	1/14 (7.1%)	4/53 (7.5%)
Heart diseases	10/39 (25.6%)	3/14 (21.4%)	13/53 (24.5%)
Lung diseases	1/39 (2.6%)	1/14 (7.1%)	2/53 (3.8%)
Previous cancer history	4/39 (10.3%)	0/14 (0.0%)	4/53 (7.5%)
Initial clinical symptoms			
Fever (°C, >37.3)	24/39 (61.5%)	7/14 (50.0%)	31/53 (58.5%)
Back pain	37/39 (94.9%)	14/14 (100.0%)	51/53 (96.2%)
Radiculopathy	21/39 (53.8%)	11/14 (78.6%)	32/53 (60.4%)
Weakness	4/39 (10.3%)	2/14 (14.3%)	6/53 (11.3%)
Bowel and bladder symptoms	1/39 (2.6%)	0/14 (0.0%)	1/53 (1.9%)
Initial radiological feature of PVO			
Extension of PVO, levels	1.44 ± 0.59 (1–3)	1.50 ± 0.52 (1–2)	1.45 ± 0.57 (1–3)
Vertebral body *	15/39 (38.5%)	12/14 (85.7%)	27/53 (50.9%)
Discitis *	22/39 (56.4%)	13/14 (92.9%)	35/53 (66.0%)
Paraspinal soft tissue	34/39 (87.2%)	14/14 (100.0%)	48/53 (90.6%)
Epidural abscess *	22/39 (56.4%)	13/14 (92.9%)	35/53 (66.0%)
Psoas abscess *	14/39 (35.9%)	11/14 (78.6%)	25/53 (47.2%)
Timing of clinical assessment and simultaneous PET/MRI, days ^a^	41.26 ± 16.29 (21–91)	43.64 ± 15.95 (21–75)	41.89 ± 16.08 (21–91)
Duration of follow-up, months ^b^	11.92 ± 7.25 (6–35)	13.43 ± 8.18 (6–29)	12.32 ± 7.45 (6–35)

Group C, cured; Group NC, non-cured; PVO, pyogenic vertebral osteomyelitis; ^a^ from starting parenteral antibiotic therapy; ^b^ from stopping parenteral antibiotic therapy; * statistical significant difference between the measures of groups C and NC; *p*-values of <0.05 were considered statistically significant.

**Table 3:** Microorganisms and antibiotics.

Factors	Values
Identification of causative bacteria	29/53 (54.7%)
Group C (*n* = 39)	20/39 (51.3%)
Group NC (*n* = 14)	9/14 (64.3%)
Causative bacteria	
Gram-positive bacteria	27/29 (93.1%)
*Staphylococcus aureus*	15/27 (55.6%)
MSSA	9/15 (60.0%)
MRSA	6/15 (40.0%)
*Coagulase-negative staphylococci*	5/27 (18.5%)
MRSE	2/5 (40.0%)
Others	3/5 (60.0%)
*Streptococcus species*	5/27 (18.5%)
*Enterococcus species*	2/27 (7.4%)
Gram-negative bacteria	2/29 (6.9%)
*Escherichia coli*	2/2(100.0%)
Non	24/53 (45.3%)
Bacterial diagnosis	
Blood	9/29 (31.0%)
PVO lesion	26/29 (89.7%)
Blood and PVO lesion	6/29 (20.7%)
Duration of parenteral antibiotics for treatment, days	45.21 ± 18.45 (21–89)
Group C (*n* = 39)	42.33 ± 17.17 (21–89)
Group NC (*n* = 14)	53.21 ± 20.13 (23–88)
Parenteral antibiotics	
β-Lactam	21/53 (39.6%)
1st generation cephalosporin	11/21 (52.4%)
3rd generation cephalosporin	3/21 (14.3%)
Nafcillin	7/21 (33.3%)
β-Lactam ± others ^a^	5/53 (9.4%)
Glycopeptide ± others ^b^	20/53 (37.7%)
Quinolone	5/53 (9.4%)
Carbapenem	2/53 (3.8%)

Group C, cured; Group NC, non-cured; MSSA, methicillin-sensitive *Staphylococcus aureus*; MRSA, methicillin-resistant *staphylococcus aureus*; MRSE, methicillin-resistant *Staphylococcus epidermidis*; PVO, pyogenic vertebral osteomyelitis; Group C, cured; Group NC, non-cured; ^a^ β-lactamase inhibitor and aminoglycoside; ^b^ Gentamicin and tazime.

**Table 4:** Comparison of clinical and radiological features between the groups C and NC.

Factors	Group C (*n* = 39)	Group NC (*n* = 14)	*p* Value	Total (*n* = 53)
**Initial**				
ESR (mm/h)	63.67 ± 29.13 (6–120)	61.79 ± 20.76 (30–98)	0.826	63.17 ± 26.99 (6–120)
CRP (mg/dL)	10.33 ± 9.33 (0.03–33.79)	8.71 ± 8.06 (0.11–28.00)	0.569	9.90 ± 8.97 (0.03–33.79)
VAS	7.51 ± 0.99 (5–9)	7.79 ± 0.89 (6–9)	0.371	7.58 ± 0.97 (5–9)
**When assessing therapeutic response**				
ESR (mm/h)	50.44 ± 29.82 (8–120) ^+^	61.79 ± 32.07 (10–120)	0.236	53.43 ± 30.54 (8–120) ^+^
CRP (mg/dL) *	1.05 ± 1.28 (0.02–5.93) ^+^	3.23 ± 3.29 (0.02–11.47) ^+^	0.030	1.63 ± 2.20 (0.02–11.47) ^+^
VAS *	4.10 ± 0.99 (2–6) ^+^	5.79 ± 1.67 (3–8) ^+^	0.003	4.55 ± 1.41 (2–8) ^+^
PvoSUV_max_ *	4.55 ± 1.43 (2.10–8.42)	7.54 ± 2.97 (3.52–14.19)	0.002	5.34 ± 2.34 (2.10–14.19)
Δ PvoSUV_max_-NmlSUV_max_ *	2.69 ± 1.44 (0.00–6.81)	5.22 ± 3.23 (1.02–12.75)	0.013	3.36 ± 2.32 (0.00–12.75)
Δ PvoSUV_max_-NmlSUV_mean_ *	3.00 ± 1.46 (0.06–7.08)	5.59 ± 3.23 (1.17–12.97)	0.011	3.69 ± 2.34 (0.06–12.97)

Group C, cured; Group NC, non-cured; ESR, erythrocyte sedimentation rate; CRP, C-reactive protein; VAS, visual analog scale; PVO, pyogenic vertebral osteomyelitis; SUV_max_, maximum standardized uptake value of ^18^F-fluorodeoxyglucose; SUV_mean_, mean standardized uptake value of ^18^F-fluorodeoxyglucose; PvoSUV_max_, SUV_max_ of PVO lesion; ΔPvoSUV_max_−NmlSUV_max_, difference between PvoSUV_max_ and SUV_max_ of normal vertebra; ΔPvoSUV_max_−NmlSUV_mean_, difference between PvoSUV_max_ and SUV_mean_ of normal vertebra; * statistical significant difference between the measures of groups C and NC; ^+^ statistical significant difference between the measures of initial and when assessing therapeutic response in each groups (*p* < 0.01); *p*-values of <0.05 were considered statistically significant.

**Table 5:** Diagnostic values of FDG-PET and CRP for residual PVO.

Factors	Cut-Off	AUC	*p* Value	Sensitivity	Specificity	PPV	NPV	DA
ESR (mm/h)	46	0.607	0.238	-	-	-	-	-
CRP (mg/dL) *	0.86	0.772	0.003	85.7%	66.7%	48.0%	92.9%	71.7%
PvoSUV_max_ *	6.44	0.833	<0.001	71.4%	89.7%	71.4%	89.7%	84.9%
ΔPvoSUV_max_-NmlSUV_max_ *	4.50	0.766	0.003	64.3%	94.9%	81.8%	88.1%	86.8%
ΔPvoSUV_max_-NmlSUV_mean_ *	4.84	0.773	0.003	64.3%	94.9%	81.8%	88.1%	86.8%
	**Cut-off**			**Sensitivity**	**Specificity**	**PPV**	**NPV**	**DA**
CRP + PvoSUV_max_	0.86 and 6.44			85.7%	94.9%	85.7%	94.9%	92.5%
CRP + ΔPvoSUV_max_-NmlSUV_max_	0.86 and 4.50			85.7%	97.4%	92.3%	95.0%	94.3%
CRP + ΔPvoSUV_max_-NmlSUV_mean_	0.86 and 4.84			85.7%	97.4%	92.3%	95.0%	94.3%

FDG-PET, ^18^F-fluorodeoxyglucose positron emission tomography; CRP, C-reactive protein; PVO, pyogenic vertebral osteomyelitis; AUC, area under the curve; PPV, positive predictive value; NPV, negative predictive value; DA, diagnostic accuracy; ESR, erythrocyte sedimentation rate; SUV_max_, maximum standardized uptake value of ^18^F-fluorodeoxyglucose; SUV_mean_, mean standardized uptake value of ^18^F-fluorodeoxyglucose; PvoSUV_max_, SUV_max_ of PVO lesion; ΔPvoSUV_max_−NmlSUV_max_, difference between PvoSUV_max_ and SUV_max_ of normal vertebra; ΔPvoSUV_max_−NmlSUV_mean_, difference between PvoSUV_max_ and SUV_mean_ of normal vertebra; * statistical significant difference between the measures of groups C and NC; *p*-values of <0.05 were considered statistically significant.

**Table 6:** Distribution-based interpretation: FDG uptake on FDG-PET, contrast enhancement of T1-weighted contrast on MRI, and high signal intensity of T2-Weighted fat saturation on MRI.

Imaging	Groups	Grade I	Grade II	Grade III	Total
FDG uptake on FDG-PET * (*p* = 0.026)	Group C	0	26	13	39
	Group NC	0	4	10	14
	Total	0	20	33	53
Contrast enhancement on T1-weighted contrast (*p* = 0.093)	Group C	0	8	31	39
	Group NC	0	0	14	14
	Total	0	8	45	53
High signal intensity on T2-weighted fat saturation * (*p* = 0.022)	Group C	0	16	23	39
	Group NC	0	1	13	14
	Total	0	17	36	53

FDG-PET, ^18^F-fluorodeoxyglucose positron emission tomography; MRI, magnetic resonance imaging; Group C, cured; Group NC, non-cured; * statistical significant difference between the measures of groups C and NC; *p*-values of <0.05 were considered statistically significant.

## References

[1] Berbari E.F., Kanj S.S., Kowalski T.J., Darouiche R.O., Widmer A.F., Schmitt S.K., Hendershot E.F., Holtom P.D., Huddleston P.M., Petermann G.W. (2015). Infectious Diseases Society of America (IDSA) Clinical Practice Guidelines for the Diagnosis and Treatment of Native Vertebral Osteomyelitis in Adults. Clin. Infect. Dis..

[2] Perronne C., Saba J., Behloul Z., Salmon-Ceron D., Leport C., Vilde J.L., Kahn M.F. (1994). Pyogenic and tuberculous spondylodiskitis (vertebral osteomyelitis) in 80 adult patients. Clin. Infect. Dis..

[3] Yu G.J., Koslowsky I.L., Riccio S.A., Chu A.K.M., Rabin H.R., Kloiber R. (2018). Diagnostic challenges in pyogenic spinal infection: An expanded role for FDG-PET/CT. Eur. J. Clin. Microbiol. Infect. Dis.

[4] Zimmerli W. (2010). Vertebral Osteomyelitis. N. Engl. J. Med..

[5] Kim J., Kim Y.S., Peck K.R., Kim E.S., Cho S.Y., Ha Y.E., Kang C.I., Chung D.R., Song J.H. (2014). Outcome of culture-negative pyogenic vertebral osteomyelitis: Comparison with microbiologically confirmed pyogenic vertebral osteomyelitis. Semin. Arthritis Rheum..

[6] Zarghooni K., Rollinghoff M., Sobottke R., Eysel P. (2012). Treatment of spondylodiscitis. Int. Orthop..

[7] Bernard L., Dinh A., Ghout I., Simo D., Zeller V., Issartel B., Le Moing V., Belmatoug N., Lesprit P., Bru J.P. (2015). Antibiotic treatment for 6 weeks versus 12 weeks in patients with pyogenic vertebral osteomyelitis: An open-label, non-inferiority, randomised, controlled trial. Lancet.

[8] Yoon S.H., Chung S.K., Kim K.J., Kim H.J., Jin Y.J., Kim H.B. (2010). Pyogenic vertebral osteomyelitis: Identification of microorganism and laboratory markers used to predict clinical outcome. Eur. Spine J..

[9] Khan M.H., Smith P.N., Rao N., Donaldson W.F. (2006). Serum C-reactive protein levels correlate with clinical response in patients treated with antibiotics for wound infections after spinal surgery. Spine J..

[10] Zarrouk V., Feydy A., Salles F., Dufour V., Guigui P., Redondo A., Fantin B. (2007). Imaging does not predict the clinical outcome of bacterial vertebral osteomyelitis. Rheumatology.

[11] Carragee E.J., Kim D., van der Vlugt T., Vittum D. (1997). The clinical use of erythrocyte sedimentation rate in pyogenic vertebral osteomyelitis. Spine.

[12] Fahnert J., Purz S., Jarvers J.S., Heyde C.E., Barthel H., Stumpp P., Kahn T., Sabri O., Friedrich B. (2016). Use of Simultaneous 18F-FDG PET/MRI for the Detection of Spondylodiskitis. J. Nucl. Med..

[13] Jeon I., Kong E. (2020). Application of Simultaneous 18F-FDG PET/MRI for Evaluating Residual Lesion in Pyogenic Spine Infection: A Case Report. Infect. Chemother..

[14] Jeon I., Kong E., Kim S.W. (2019). Simultaneous 18F-FDG PET/MRI in tuberculous spondylitis: An independent method for assessing therapeutic response—case series. BMC Infect. Dis..

[15] Nanni C., Boriani L., Salvadori C., Zamparini E., Rorato G., Ambrosini V., Gasbarrini A., Tumietto F., Cristini F., Scudeller L. (2012). FDG PET/CT is useful for the interim evaluation of response to therapy in patients affected by haematogenous spondylodiscitis. Eur. J. Nucl. Med. Mol. Imaging.

[16] Riccio S.A., Chu A.K., Rabin H.R., Kloiber R. (2015). Fluorodeoxyglucose Positron Emission Tomography/Computed Tomography Interpretation Criteria for Assessment of Antibiotic Treatment Response in Pyogenic Spine Infection. Can. Assoc. Radiol. J..

[17] Russo A., Graziano E., Carnelutti A., Sponza M., Cadeo B., Sartor A., Righi E., Bassetti M. (2019). Management of vertebral osteomyelitis over an eight-year period: The UDIPROVE (UDIne PROtocol on VErtebral osteomyelitis). Int. J. Infect. Dis..

[18] Kim S.J., Kim I.J., Suh K.T., Kim Y.K., Lee J.S. (2009). Prediction of residual disease of spine infection using F-18 FDG PET/CT. Spine.

[19] Kowalski T.J., Berbari E.F., Huddleston P.M., Steckelberg J.M., Osmon D.R. (2006). Do follow-up imaging examinations provide useful prognostic information in patients with spine infection?. Clin. Infect. Dis..

[20] Lora-Tamayo J., Euba G., Narvaez J.A., Murillo O., Verdaguer R., Sobrino B., Narvaez J., Nolla J.M., Ariza J. (2011). Changing trends in the epidemiology of pyogenic vertebral osteomyelitis: The impact of cases with no microbiologic diagnosis. Semin. Arthritis Rheum..

[21] Flury B.B., Elzi L., Kolbe M., Frei R., Weisser M., Scharen S., Widmer A.F., Battegay M. (2014). Is switching to an oral antibiotic regimen safe after 2 weeks of intravenous treatment for primary bacterial vertebral osteomyelitis?. BMC Infect. Dis..

[22] Goldsmith S.J., Vallabhajosula S. (2009). Clinically proven radiopharmaceuticals for infection imaging: Mechanisms and applications. Semin. Nucl. Med..

[23] Signore A., Glaudemans A.W. (2011). The molecular imaging approach to image infections and inflammation by nuclear medicine techniques. Ann. Nucl. Med..

[24] Bjorksten B., Boquist L. (1980). Histopathological aspects of chronic recurrent multifocal osteomyelitis. J. Bone Jt. Surg. Br. Vol..

